# The potential effects of an extended alcohol withdrawal treatment programme on morbidity and mortality among inpatients in the German city of Bremen: a simulation study

**DOI:** 10.1186/s13011-019-0249-7

**Published:** 2020-01-02

**Authors:** Jakob Manthey, Christina Lindemann, Ludwig Kraus, Jens Reimer, Uwe Verthein, Bernd Schulte, Jürgen Rehm

**Affiliations:** 1 Institute of Clinical Psychology and Psychotherapy, Technische Universität Dresden, Chemnitzer Str. 46, 01187 Dresden, Germany; 2 Center for Interdisciplinary Addiction Research (ZIS), Department of Psychiatry and Psychotherapy, University Medical Center Hamburg-Eppendorf, Hamburg, Germany; 3 IFT Institut für Therapieforschung, Munich, Germany; 4 Gesundheit Nord, Bremen, Germany; 5 Institute for Mental Health Policy Research, Centre for Addiction and Mental Health, Toronto, Canada; 6 Dalla Lana School of Public Health, University of Toronto, Toronto, Canada; 7 Department of Psychiatry, University of Toronto, Toronto, Canada; 8 Department of International Health Projects, Institute for Leadership and Health Management, I.M. Sechenov First Moscow State Medical University, Moscow, Russian Federation

**Keywords:** Alcohol, Treatment, Withdrawal, Detoxification, Alcohol use disorders, Hospital, Guidelines, Germany

## Abstract

**Background:**

According to the German guidelines, people with severe alcohol use disorders (AUDs) should receive withdrawal treatment. Compared to somatic withdrawal treatment (SWT), extended duration and psychosocial elements of so-called “qualified withdrawal treatment” (QWT) aim to reduce relapse rates. Despite promising results of prospective studies on QWT, only few German inpatients seeking withdrawal treatment receive QWT. We estimated the potential effects on mortality and morbidity for higher proportions of treatment-seeking patients receiving QWT rather than SWT in the German city of Bremen.

**Methods:**

In 2016 and 2017, 2051 inpatients were admitted to two specialised hospitals for withdrawal treatment. The potential beneficial effects of QWT over SWT were estimated by simulating treatment outcomes taken from two prospective studies. Outcomes comprised number and length of all-cause hospitalisations within 5 years, as well as abstinence and all-cause mortality rates within 28 months. Outcomes were estimated for actual and increased rates of QWT (25, 50%) among inpatients seeking alcohol treatment.

**Results:**

In the selected hospitals, 170 patients (8%) received QWT. If 25% of AUD inpatients were to receive QWT, benefits in abstinence rates (+ 18%), the total number of hospitalisations (− 9%) and hospital days (− 10%) could be expected. If 50% of AUD inpatients were to receive QWT, benefits in abstinence rates (+ 45%), the total number of hospitalisations (− 23%) and hospital days (− 26%) were more pronounced, in addition to reductions in mortality (− 20%).

**Conclusion:**

Increasing the proportion of people with severe AUD enrolled in extended withdrawal treatment programs (such as QWT) may contribute to reduce overall alcohol-attributable burden of disease. Randomised controlled trials or other prospective studies controlling for confounding factors are needed to determine the potential at the population level.

## Background

In Europe, both alcohol consumption and attributable burden remain at a high level, albeit with decreases in past years [1, 2]. Alcohol control policies to further reduce alcohol-attributable burden in Europe are manifold and include taxation and minimum pricing [3, 4], alcohol monopoly [5], and restrictions in marketing and availability [6]. Aside from alcohol control policies, alcohol treatment programs have also received a lot of attention, primarily in the form of screening and brief interventions, which were found to have small but relevant public health effects if implemented widely [6–8].

In Europe, 71% of the alcohol-attributable burden stems from alcohol dependence which made up only 3.5% of the adult population [9]. However, some of the proposed measures to reduce alcohol-attributable harm, including the so called best buys declared by the World Health Organization (WHO) [10], do not target people with alcohol use disorders (AUDs) in particular or are even less effective in this population. For instance, people with severe AUDs are recommended to be referred to specialists rather than being offered a brief intervention [11, 12] and people with heavy drinking levels were found to respond less to price increases than those with moderate drinking levels [13]. Consequently, measures to reduce mortality and morbidity among people with AUDs are warranted.

In 2016, 8.8% of the adult population were estimated to have an AUD in the WHO European Region [10]. There is a consensus that AUDs are generally underdiagnosed in the health care system and treatment coverage is comparably low, with about 1 in 10 people in need for treatment receiving some form of treatment in European countries [10, 14–16]. In one of the few studies modelling the impact of AUD interventions, it was estimated that a 40% treatment coverage rate of evidence-based interventions (i.e. motivational interviewing, cognitive behavioural therapy, brief interventions, and pharmacological interventions) would prevent nearly 12,000 alcohol-attributable deaths in the European Union in 2004 [9].

In general, interventions offered to patients with AUD should depend on the individual needs, their drinking levels and alcohol problems and further determined by provider capacities and health care system properties [17, 18]. For people with more severe AUDs or higher drinking levels, acute care should primarily aim at treating withdrawal symptoms as well as complications arising from comorbidities, usually with pharmacotherapy [19, 20]. Further, in order to mitigate the risk of relapse after withdrawal treatment and to stabilize patients, continuous psychosocial interventions should be offered. This scheme is reflected in the United Kingdom NICE Pathway on Assisted alcohol withdrawal [21] and also in the German Guideline on Screening, Diagnosis and Treatment of Alcohol Use Disorders, which were developed based on available evidence on diagnostics and treatment of alcohol-related disorders [22]. According to the NICE pathway, an intensive community programme over a three-week period should be offered for persons with moderate AUD and complex needs or those with severe AUD. In contrast, the German guideline state that so-called “qualified withdrawal treatment” (QWT) should be offered to all people instead of the somatic withdrawal treatment (SWT). SWT is a short-term (3 to 5 days) out- or inpatient program, which aims to support the alcohol detoxification and withdrawal-related symptoms with pharmacotherapy. SWT intends to stabilize the medical conditions of patients and to prevent further complications such as seizures or cardiovascular problems. In contrast, the QWT includes pharmacological interventions for withdrawal symptoms but further adds psychosocial interventions to increase the patients’ willingness to change and to stabilize abstinence within a minimum duration of 3 weeks, which can be delivered in both inpatient and outpatient settings [20]. Besides detoxification, one main goal of QWT is to stabilize patients’ self-esteem, to create a confident atmosphere which aims to facilitate the motivation to give up drinking. In general, QWT contains elements of psychotherapy including group therapy, and the treatment of co-morbidities. Furthermore, part of QWT is delivering information on further treatment options such as medical rehabilitation in in−/outpatient settings or autonomous self-help groups.

According to the German guidelines, all people with AUDs should be offered withdrawal treatment, which should be provided in inpatient settings for patients at risk for developing withdrawal syndrome. Further, QWT is unequivocally recommended over SWT in the German guideline, however, the literature on patient outcomes comparing QWT and SWT is limited. To our knowledge and based on the systematic literature review undertaken for drafting the guidelines, there are only three prospective studies which followed up patients with AUD between 2 months and 5 years after receiving QWT or SWT. According to their results, QWT was superior to SWT in terms of number and length of hospitalizations [23], abstinence [24, 25], as well as survival rates [25]. Despite these promising findings, the number of patients with AUD receiving QWT in Germany remains low [26]. In this simulation study, we increased the proportion of patients receiving QWT in a sample of inpatients seeking withdrawal treatment in the German city of Bremen and examined potential effects on mortality and morbidity.

## Methods

### Data sources

Data on hospital admissions of persons with a main diagnosis of any alcohol-related disorder (F10 diagnoses according to the 10th Revision of the International Classification of Diseases [27]) in 2016/2017 were obtained from those two hospitals exclusively offering alcohol withdrawal treatment in the city of Bremen (Germany). In these hospitals, both SWT and QWT is offered to the patients. Each patient will be thoroughly informed about the implications of each treatment program but the decision will eventually rest with the patient only.

The simulation draws on parameters taken from two prospective studies conducted between 1989 and 1997. In the first study, all patients admitted to the hospital clinic for alcohol withdrawal treatment in Lübeck, Germany, between August 1989 and March 1991, who were insured with a regional statutory health insurance were included for a register-based study, resulting in a sample of 180 patients (*n* = 79 SWT; *n* = 101 QWT) [23]. The study does not provide any details on sociodemographic or other potentially confounding variables but describes various reasons for loss to follow-up (discontinued membership with health insurance, death, not located), with the final sample including 94 patients (*n* = 37 SWT; *n* = 57 QWT). The statutory health insurance provided data on hospitalisations over 5 subsequent years.

In the second study, all 182 patients (*n* = 90 SWT; *n* = 92 QWT) treated in 1994 in a hospital clinic in Jena, Germany, were sampled and followed up for 28 months on average [25]. At baseline, this sample was on average 42 years old, consisted of 22% females and had a history of alcohol dependence of 11.3 years on average. While these indicators did not statistically differ between both groups, the SWT patients reported lower daily alcohol intake levels (220 vs 305 g pure alcohol per day), had fewer comorbidities, were more likely to be admitted to hospital via emergency room, and had lower educational achievements, as compared to the QWT group. The dropout rates did not differ between both groups (SWT: 30.0%, QWT: 30.4%) and the final sample included 127 patients (*n* = 63 SWT; *n* = 64 QWT).

As summarized in Table 1, we obtained simulation parameters on A) number and B) length of all-cause hospitalisations from the first study (within a follow-up interval of 5 years, [23]), and parameters on C) abstinence and D) all-cause mortality rates from the second study (follow-up interval of 28 months, [25]).

**Table 1:** Data required for simulating AUD treatment outcomes

Study	Sample size at baseline (lost to follow-up)^a^	Outcome		Mean outcome in QWT vs SWT group	Dispersion parameters for negative binomial distribution
Driessen et al., 1999 [25]	n in SWT = 79 (42)	A)	Number of hospitalisations within 5 years	3.5 (4.4)^b^ vs. 7.3 (11.3)^b^	QWT: 0.774
					SWT: 0.443
	n in QWT = 101 (44)				
		B)	Length of hospitalisations within 5 years	55.7 (75.4)^b^ vs. 135.8 (167.3)^b^	QWT: 0.551
					SWT: 0.662
Bauer et al., 2000 [23]	n in SWT = 90 (40)	C)	Proportion abstinent within 28 months	31.5% (29/92)^b^ vs. 14.4% (13/90)^b^	N/A
	n in QWT = 92 (35)				
		D)	Proportion dead within 28 months	7.6% (7/92)^b^ vs. 14.4% (13/92)^b^	N/A

Note. *AUD* Alcohol use disorder, *QWT* Qualified withdrawal treatment, *SWT* Somatic withdrawal treatment; *N/A* Not applicable

^a^ Patients lost to follow-up (i.e., could not be contacted) were not included in calculation of the means and standard deviation for indicator A) and B) and were regarded as non-abstinent and not dead for outcomes C) and D), respectively

^b^ Numbers in brackets indicate standard deviation (continuous variables) or the numerator and denominator (binary variables)

### Statistical analyses

In this simulation study, we extrapolated the trajectories reported in previous studies. For this purpose, we assumed that the current sample of patients with AUD recruited in 2016/2017 would follow the same trajectories for abstinence, morbidity, and mortality as patients with AUD did in previous, prospective studies (see Table 1). For each outcome A-D, we ran the following scenarios: 1) proportion of patients receiving QWT as reported by the hospital (baseline), 2) 25% of patients receiving QWT, 3) 50% of patients receiving QWT. In the two hypothetical scenarios 2) and 3), the group size of patients receiving QWT was increased, with an equivalent reduction of the number of patients receiving SWT, while the total N remained constant.

For each scenario, group and outcome, 10,000 sample distributions were drawn. For continuous variables (number of hospitalisations, number of inpatient days), the outcome of interest was determined by sampling from negative binomial distributions, which allows to model right skewed count variables, such as hospitalisations and number of inpatient days [28]. All required parameters for the negative binomial distribution could be directly obtained from the figures reported in Table 1, except for the dispersion parameter, which was calculated from the mean and variance (dispersion = mean^2 / (−mean + variance), see Table 1).

For binary variables (abstinence, mortality), the outcome of interest was determined by sampling from binomial distributions [29]. All required parameters for the binomial distribution could be directly obtained from the figures reported in Table 1.

To give an example for the baseline scenario, the number of abstinent persons after 28 months in the QWT group was obtained from a binomial distribution of all patients in this group. A sample distribution was drawn using the probability of abstinence at follow-up (see Table 1). This sampling was repeated 10,000 times to obtain the mean and the 95% confidence interval of the number of people abstinent in this group. To obtain the total number of abstinent patients at follow-up in this scenario, the same procedure was repeated for patients in the SWT group and results of both groups were combined.

Eventually, the variables in the different scenarios (baseline, 25, 50%) were compared using the 10,000 sampled estimates. For example, the difference in the number of abstinent people was calculated as the mean difference between both scenarios across all 10,000 samples. Using these differences, the 95% confidence interval was constructed, as well.

All data were processed and analysed using R version 3.5.1 [30]. The full R code to reproduce all findings is enclosed as Additional file 1.

## Results

In 2016 and 2017, a total of 2051 people with AUD (26.2% females) had been admitted as inpatients to two German hospitals. The most frequent main diagnosis was “withdrawal state” (F10.3, 53.5%), followed by “dependence syndrome” (F10.2, 38.6%), “acute intoxication” (F10.0, 5.5%), and “harmful use” (F10.1, 2.3%). Across all patients, 170 (8%) received QWT (baseline scenario). According to our models, a total number of around 14,000 hospitalisations would accumulate over a period of 5 years, resulting in about 265,000 days spent in hospital. Further and within 28 months after treatment, we estimated that 384 (16%) of all patients would remain abstinent and another 262 (14%) would die.

In the alternative scenario, where 25% (*n* = 513) inpatients would receive QWT, benefits in morbidity and abstinence could be expected, as compared to the baseline scenario. Specifically, we estimate that an additional 58 patients could remain abstinent (+ 18%) and the total number of hospitalisations and hospital days could be curbed by about 1300 (− 9%) and 27,000 (− 10%), respectively. For the estimated reductions in mortality, the confidence intervals overlapped with 0, indicating no meaningful change from baseline.

For the additional scenario, in which half of the patients would receive QWT, the modelled benefits were even more pronounced. As compared to the baseline scenario, an additional 146 patients could remain abstinent (+ 45%), and the total number of hospitalisations and hospital days could be reduced by about 3200 (− 23%) and − 69,000 (− 26%), respectively. Further, if 50% of patients received QWT, 59 fewer deaths (− 20%) could be expected.

**Fig. 1:**
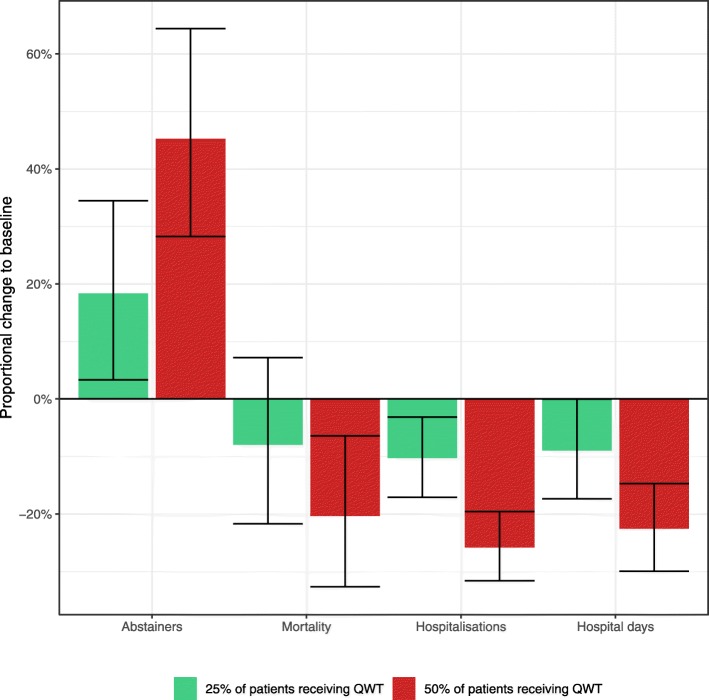
Proportional change in treatment outcomes under two different scenarios of patients receiving QWT (25%/50%), as compared to baseline (8%). Green bar = scenario in which 25% of inpatients received QWT; Red bar = scenario in which 50% of inpatients received QWT; error bars indicate 95% confidence intervals

The mean estimates for each scenario, as well as the proportional changes to baseline are also presented in Table 2 and Fig. 1.

**Table 2:** Simulated outcomes for three different scenarios based on 2051 inpatients admitted for alcohol withdrawal treatment

	Baseline scenario (8% of patients receiving QWT)	25% of patients receiving QWT		50% of patients receiving QWT	
	n (QWT) = 170	n (QWT) = 513		n (QWT) = 1026	
	n (SWT) = 1881	n (SWT) = 1538		n (SWT) = 1025	
	Mean	Mean	Difference to baseline in %	Mean	Difference to baseline in %
Number of hospitalisations within 5 years	14,325 (13,378 to 15,316)	13,022 (12,139 to 13,929)	−9% (−17 to −0.02%)	11,076 (10,336 to 11,851)	−23% (−30 to −15%)
Number of days spent in hospital within 5 years	264,849 (250,609 to 279,444)	237,394 (224,235 to 250,681)	−10% (−17 to − 3%)	196,331 (184,922 to 208,170)	−26% (−32 to −20%)
Proportion abstinent within 28 months	16% (14 to 17%)	19% (17 to 20%)	+ 18% (3 to 34%)	23% (21 to 25%)	+ 45% (28 to 64%)
Proportion dead within 28 months	14% (12 to 15%)	13% (11 to 14%)	−8% (−22 to 7%)	11% (10 to 12%)	−20% (−33 to −6%)

Note. *QWT* Qualified withdrawal treatment, *SWT* Somatic withdrawal treatment

Numbers in brackets represent 95% confidence intervals

## Discussion

In this contribution, we simulated the potential effects of an extended alcohol withdrawal treatment program on abstinence, morbidity and mortality using data from two hospitals in Bremen, Germany. According to our tentative estimations, mortality and morbidity could be cut by one fourth and one fifth in the studied sample, respectively, if every second patient with AUD seeking withdrawal treatment was to enroll in an extended withdrawal program and if the previously conducted studies can be generalized.

In the European region, alcohol-attributable disease burden remains high, with nearly 800,000 deaths in 2016 [2]. Efforts to reduce this burden need to be manifold and AUD treatment has been acknowledged as important aspect in the Global Strategy to reduce the harmful use of alcohol [31]. Clearly, measures to increase the number of people with AUD to receive treatment, e.g. via routine alcohol screenings in primary health care and hospitals are warranted [12]. However, our findings add that the potential of improving interventions for those already seeking treatment should be considered and elaborated in future studies.

In 2011, only one in three European countries provided national guidelines on AUD treatment [32], with unknown specifications of withdrawal treatment. It remains unclear how withdrawal treatment is routinely implemented in European countries, which were found to largely vary with respect to health care systems [18]. In this contribution, we show that less than one in ten patients admitted to two German hospitals for withdrawal treatment have received optimal interventions, i.e. QWT, as recommended by the German guidelines. As compared to previous studies in the field, this share appears to be comparably low [23, 25].

Although literature concerning the barriers of QWT for patients with AUD is sparse, we expect barriers to be located within the patient, the providers, and the health insurance. For the patient, the main barrier may be the fear of being stigmatized for receiving treatment for AUD, which has been identified as important barrier for AUD treatment in general [33]. As QWT requires a longer hospital stay, the longer absence from workplace or other activities may increase the likelihood of alcohol problems to be recognized by colleagues, family and friends. Furthermore, some providers may not recognize and thus recommend QWT as the best available treatment option, which would not be surprising given the lack of awareness of guideline content among many German physicians (Frischknecht et al.: Wer screent wen? Ergebnisse einer Versorgerbefragung zum Screening auf problematischen Alkoholkonsum im Bundesland Bremen, in preparation). Lastly, QWT may not be routinely covered by the statutory health insurance, especially if two withdrawal treatment episodes (i.e., short period of relapse) follow with a short period of time, posing a rather structural barrier to provide more QWT in this population.

Further, an international harmonization of terms and concepts related to withdrawal treatment should be undertaken. In Germany, the term QWT is well-known among clinicians whereas in the UK, “assisted withdrawal treatment” appears to be a concept with large overlaps to QWT [21]. Recommendations of psychosocial interventions following acute withdrawal treatment can also be found elsewhere (e.g., see [34]), however without details on the type and duration of interventions being specified. We are not aware of other terms describing extended withdrawal treatment programs, which encompass psychosocial interventions over a duration of several weeks.

Lastly, more studies are required to capture the full potential of an extended withdrawal programs for reducing the alcohol-attributable burden of disease. Most importantly, prospective studies controlling for selection biases are required to reliably investigate the effects on abstinence, drinking levels, mortality and morbidity. Furthermore, to assess the feasibility of widening extended withdrawal programs among patients with severe AUD seeking treatment, studies should examine the cost-effectiveness of those programs. Previously, hospital stays among AUD patients nearly doubled those of other patients, making up substantial shares of health care costs attributable to AUD [35]. It remains to be determined if the benefits in terms of fewer hospitalizations and fewer deaths can outweigh the costs arising from an extensive withdrawal treatment programs, which do not necessarily need to be conducted in inpatient settings, as recommended in the UK guidelines.

### Limitations

The estimates presented in this study should be interpreted with caution as the simulation parameters were taken from prospective studies that did not fully control for potential confounders. In one study providing simulation parameters, both SWT and QWT patients were similar in most confounding variables [25], however, the other study did not provide a description of the sociodemographic variables [23]. As patients were not randomly assigned to SWT or QWT, we cannot exclude that different trajectories in the presented outcomes are confounded by self-selection bias. Further, since both studies are rather old, the simulated health outcomes may not represent current outcomes of AUD patients after withdrawal treatment, which may be characterized by improved health care provision and reduced risk of dying. Thus, the simulated effects on morbidity and mortality may actually be misestimated. Moreover, the number of prospective studies comparing SWT and QWT on outcomes of interests are limited, which did not allow to summarize effect sizes through meta-analyses. Adequately designed studies controlling for potential confounders, such as randomized controlled trials, are required for a better understanding and to overcome these limitations. Lastly, our analyses are limited to inpatient treatment only, however, QWT can as well be delivered in outpatient settings.

## Conclusion

Extrapolating from two prospective studies conducted in the 1990s, this simulation study tentatively demonstrates the potential benefits of an extended withdrawal program for persons with AUD seeking inpatient treatment. Improvement of AUD treatment should be considered as one component in reducing alcohol-attributable burden of disease, in addition to alcohol policies that do not target people with AUD specifically.

## Supplementary information

**Additional file 1.** R code to reproduce all findings reported in this study. (R 20 kb)

## Data Availability

All data generated or analysed during this study are included in this published article and its supplementary information files.

## Abbreviations

AUDs: Alcohol use disorders
QWT: Qualified withdrawal treatment
SWT: Somatic withdrawal treatment
WHO: World Health Organization
